# The risk of heart-specific death in breast cancer patients

**DOI:** 10.1038/s41598-025-12648-6

**Published:** 2025-08-02

**Authors:** Yunbo Luo, Long Zhao, Peng Qu, Shiqi Han, Yali Wang, Xue Li, Jun Liu, Cui Ma, Shishan Deng, Qi Liang, Lingmi Hou, Panke Cheng

**Affiliations:** 1 Department of Breast Surgery, Plastic Surgery, Sichuan Cancer Hospital & Institute, Sichuan Cancer Center, School of Medicine, University of Electronic Science and Technology of China, Chengdu, 610041 Sichuan P.R. China; 2 Department of Academician (expert) Workstation, Biological Targeting Laboratory of Breast Cancer, Breast and Thyroid Surgery, Affiliated Hospital of North Sichuan Medical College, Nanchong, 637000 Sichuan P.R. China; 3 Department of Neurosurgery, Affiliated Hospital of North Sichuan Medical College, Nanchong, 637000 Sichuan P.R. China; 4 Department of Clinical Laboratory, Affiliated Hospital of North Sichuan Medical College, Nanchong, 637000 Sichuan P.R. China; 5 School of Laboratory Medicine, North Sichuan Medical College, Nanchong, 637007 Sichuan P.R. China; 6 Translational Medicine Research Center, North Sichuan Medical College, Nanchong, 637007 Sichuan P.R. China; 7 Institute of Basic Medicine and Forensic Medicine, North Sichuan Medical College, Nanchong, 637000 Sichuan P.R. China; 8 Institute of Cardiovascular Diseases, Department of Cardiology, School of Medicine, Sichuan Provincial People’s Hospital, University of Electronic Science and Technology of China, Chengdu, 610072 P.R. China; 9 Department of Mathematics, Army Medical University, Chongqing, 400038 P.R. China; 10 Ultrasound Medicine and Computational Cardiology Key Laboratory of Sichuan Province, Chengdu, 610072 P.R. China; 11 No.32, West 2nd Section, 1 st Ring Road, Chengdu, 610072 Sichuan P.R. China; 12 No.55, Section 4, South Renmin Road, Wuhou District, Chengdu, 610041 Sichuan P.R. China; 13 No. 1 South Maoyuan Road, Shunqing District, Nanchong, 637000 Sichuan P. R. China

**Keywords:** Cancer, Breast cancer, Cancer therapy

## Abstract

With the improvement of comprehensive anti-cancer treatment for breast cancer (BC), more and more BC survivors will die from non-cancer diseases, including cardiovascular disease dominated by heart disease (HD). Therefore, this study aimed to analyze the risk of heart-specific death (HSD) in patients with BC by using the Surveillance, Epidemiology, and End Results (SEER) database. The eligible patients diagnosed with BC between 2000 and 2019 were exported from the SEER database. The standard mortality ratios (SMR) were calculated to compare the difference in HSD between patients with BC and the general population. The Cox Proportional hazard model was used to estimate the risk factors for HSD in BC patients and the 95% confidence intervals (CI) were calculated. Overall, 655,552 eligible patients were included in our study, and 149,708 (22.8%) patients died. Among the deaths, 22,718 (15.2%) cases were attributed HD which was the second cause of death for BC patients. With the extension of follow-up (> 10 years), HD surpassed breast cancer as the leading cause of death for BC (22.3% vs. 20.2%). The SMR for HD was 8.14 (95%CI: 8.04–8.25) in the whole cohort. Multivariate analysis showed that race, age, marital status, median household income, grade, stage and subtype were independent risk factors for HSD in BC patients. The risk of HSD is significantly higher in BC patients than in the general population and closely related to demographic characteristics and tumor clinicopathological factors. Medical approaches are needed to reduce the risk of HD among patients with BC.

## Introduction

Breast cancer (BC) is the most common malignant tumor among women and poses a serious threat to women’s life and physical health^1^. Fortunately, the diagnosis and treatment of breast cancer has been improved greatly in recently years. Due to the popularity of breast cancer-related screening, more and more early-stage breast cancers are being detected, which significantly improves the effectiveness of breast cancer treatment^2^. In addition to the early diagnosis, advances in breast cancer treatment have also contributed to reducing the risk of death for patients with BC. Recently, a research conducted by Stanford University School of Medicine showed that the mortality rate declined by 58% absoltely in 2019 compared with 1975, especially a 71% decline in patients with estrogen receptor-positive (ER+)/human epidermal growth factor receptor 2-positive (HER2+) BC^3^. Another study from Northern European countries also showed that the long-term survival rate of BC patients had improved significantly, with 5-year and 10-year survival rates of 92.3% and 87.8%, respectively^4^. In China, a retrospective study showed that the 5-year and 10-year overall survival rates were 92.9% and 87.4% for patients with operable BC^5^. Thus, more and more patients with BC will die from other diseases instead of BC, which has arisen wide attentions from oncologists. Cardiovascular disease (CVD) is the most common cause of death in the global population^6^, and it also seriously compromises the long-term survival for patients with BC. A large cohort studies have shown that CVD has surpassed breast cancer as the leading cause of death with the extension of survival for patients with early BC^7^. Also, another analysis of Surveillance, Epidemiology, and End Results (SEER) database showed that CVD was the leading cause of death among older women with breast cancer (15.9% [95% CI 15.6 to 16.2]), followed by breast cancer (15.1% [95% CI 14.8 to 15.4])^8^. Thus, the prevention and treatment of CVD will become the breakthrough point for improving the long-term survival of patients with BC.

CVD is a major category of diseases including heart disease (HD), hypertension, cerebrovascular disease, macrovascular disease, peripheral vascular disease and etc. HD is the most common and fatal type of CVD^9^, which includes coronary artery disease, heart failure, arrhythmias, congenital heart defects, valvular heart diseases, and other diseases related to heart. However, those subtypes were used as an overall outcome event to analyze the risk of death among BC patients in previous studies^8–10^. In fact, there are significant differences in the disease characteristics, clinical progression, and survival outcomes among the various subtypes of CVD. For example, while HD is often associated with coronary artery disease, myocardial infarction, and heart failure, other forms of CVD, such as cerebrovascular diseas, peripheral vascular disease, and hypertension, involve distinct pathophysiological processes and risk factors. These differences can lead to varying prognoses, treatment responses, and outcomes in patients. As a result, grouping all types of CVD together when analyzing the risk of death among breast cancer (BC) patients fails to provide an accurate estimation of the specific risk posed by HD in this population. Each subtype of CVD has its own unique impact on survival, and overlooking these distinctions may obscure the true risk associated with HD. Therefore, the aim of our study was to analyze the risk of death causing by HD and the risk factors among patients with BC by using the SEER database.

## Materials and methods

### Data source

The Surveillance, Epidemiology, and End Results (SEER) Program covers approximately 48% of the US population and provides information on cancer statistics about comprehensive demographic and cancer-specific information, such as demographics, primary tumor site, tumor morphology and stage at diagnosis, first course of treatment. Additionally, the follow-up data include vital status, and the latest follow-up date for the 2020 submission is December 31, 2019. Moreover, the standardized incidence/mortality ratios can be generated by the SEER*Stat software. Our study was conducted in accordance with the Declaration of Helsinki (as revised in 2013).

#### Patient selection

**Fig. 1:**
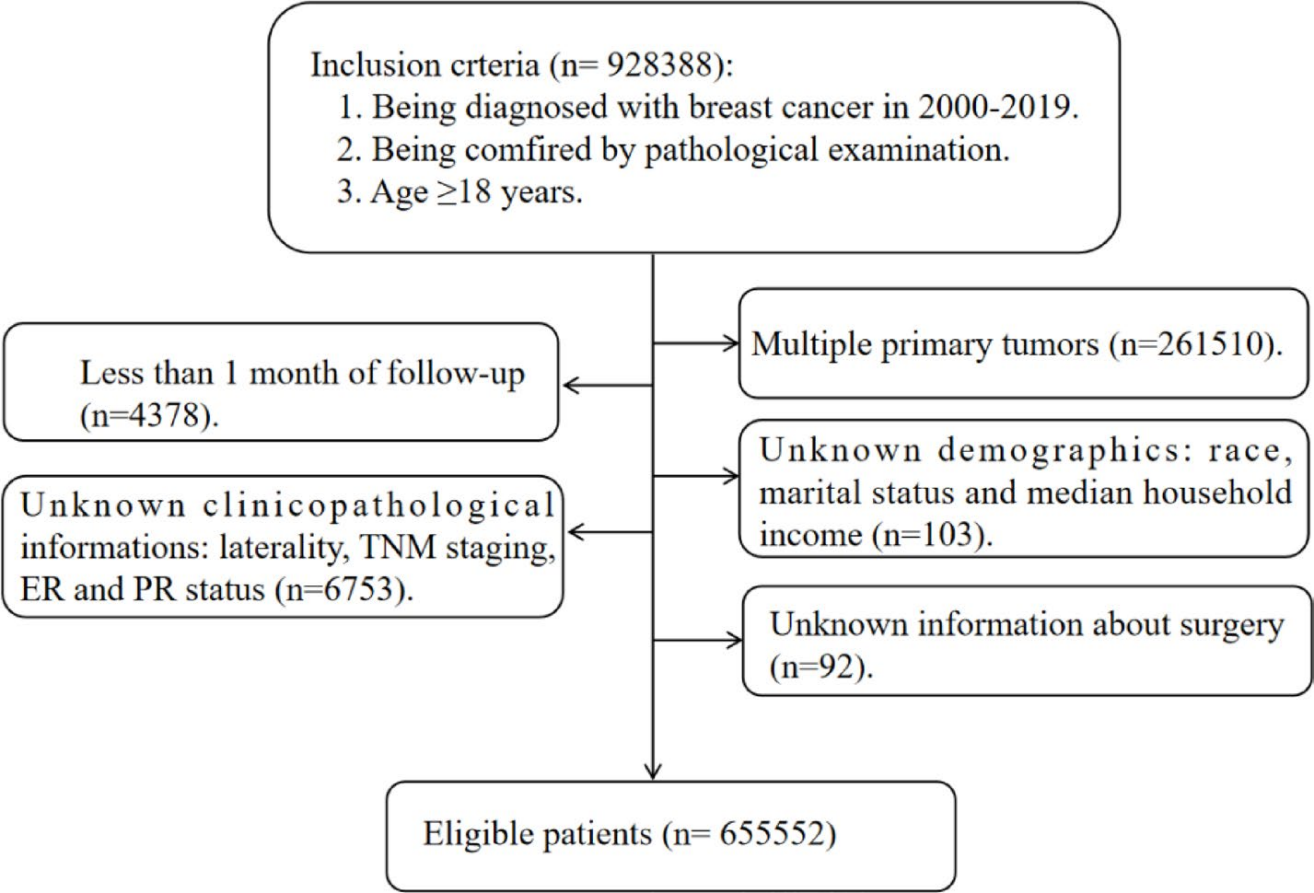
Flowchart for patient selection from the Surveillance, Epidemiology and End Results (SEER) database. ER, estrogen receptor; PR, progesterone receptor.

The patients with BC were extracted from the SEER database according the following inclusion criteria: being diagnosed with BC from 2000 to 2019; being confirmed by pathological examination; at least 18 years old. Then, the exclusion criteria were the following: patients with other malignant tumors; less than 1 month of follow-up; patients with unknown demographics, such as race, marital status and median household income; patients with unknown clinicopathological information, such as laterality, TNM staging, ER and PR status; and patients with unknown information about surgery. Finally, 655,552 cases with BC met the criteria and were analysed in our study (Fig. 1). Our study was approved by our Ethics Committee to be exempt from ethical review because the patient’s personally identifiable information could not be obtained from the SEER database.

##### Statistical analysis

Descriptive statistics were used to summarize data, including median for continuous data, and frequency and percentage for categorical data. Proportional mortality ratio (PMR) was defined as the number of deaths attributable to a specific cause divided by the total number of deaths. Standardized mortality ratios (SMR) were used to estimate the risk of death for patients with BC compared with the general population and the corresponding 95% confidence intervals (CI) were calculated. SMR was defined as the ratio of the actual mortalities due to a specific cause in our study group to expected mortalities in general US population. The expected deaths were estimated by multiplying person-years at risk in the study group by mortality rate in general population of the same age and sex group during the same period. Patients were categorized into different outcome events based on the causes of death (COD) to site recode recorded in the SEER database. Heart-specific death (HSD) was defined as the death from HD for patients with BC and heart-specific survival (HSS) referred to the interval from the diagnosis of breast cancer to death caused by HD or the final follow-up in censored cases. The Cox proportional hazards model was used to estimate the effect of variables on HSS and hazard ratios (HR) with 95% CI were calculated. Then, the multivariable Cox proportional hazards model was used estimate the independent risk factors for HSD. STATA software (Version 13; Stata Corporation) was applied for all statistical analyses and the figures were generated by Microsoft Office Excel (Version 2021 Microsoft Corporation). All tests were two sided and p value < 0.05 were considered statistically significant.

## Results

### Patient demographics

**Fig. 2:**
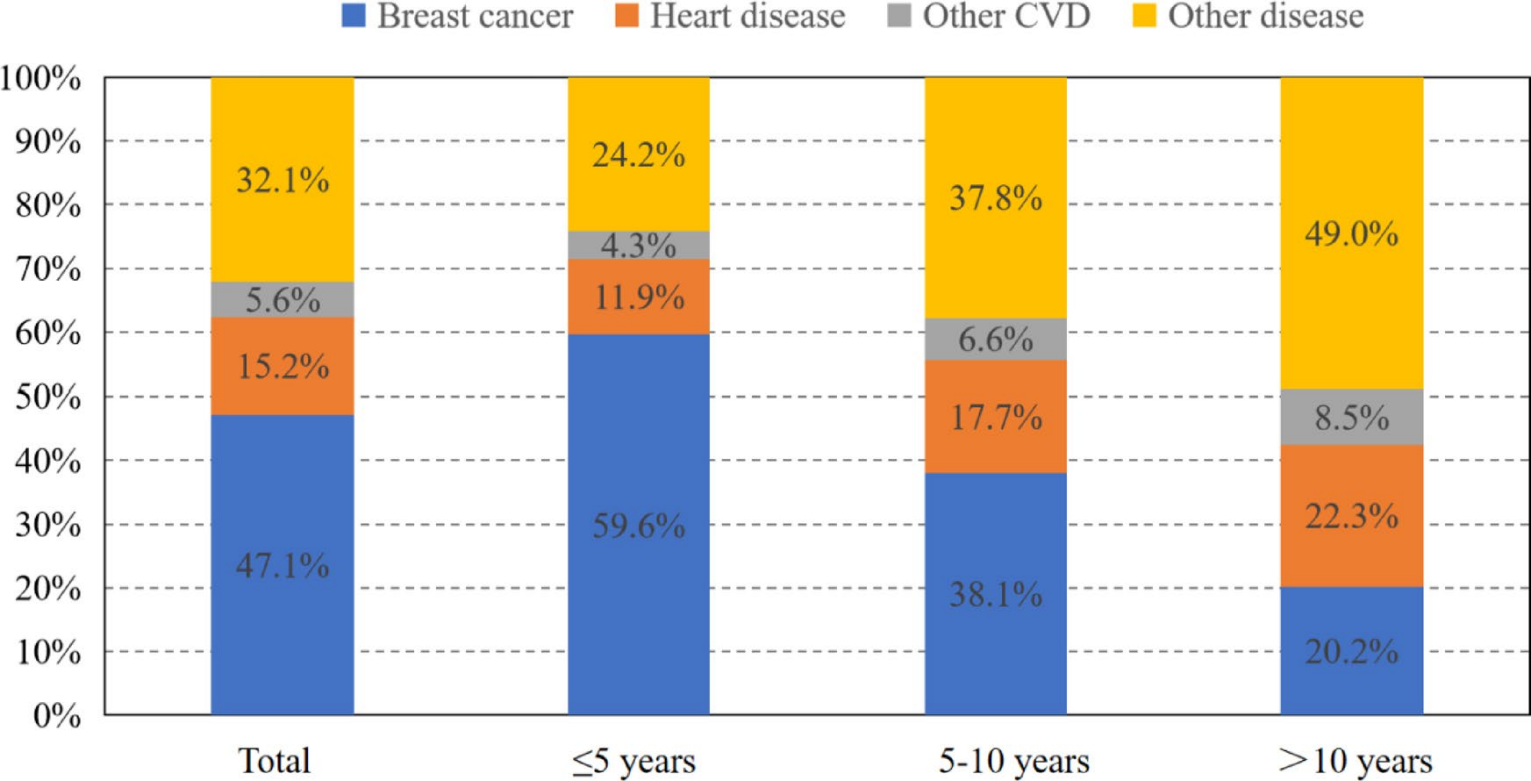
Causes of death in each latency period following breast cancer diagnosis. CVD, cardiovascular disease. Other CVD refers to conditions such as cerebrovascular disease, peripheral artery disease, arrhythmias, and other related disorders, excluding heart disease (HD).

**Fig. 3:**
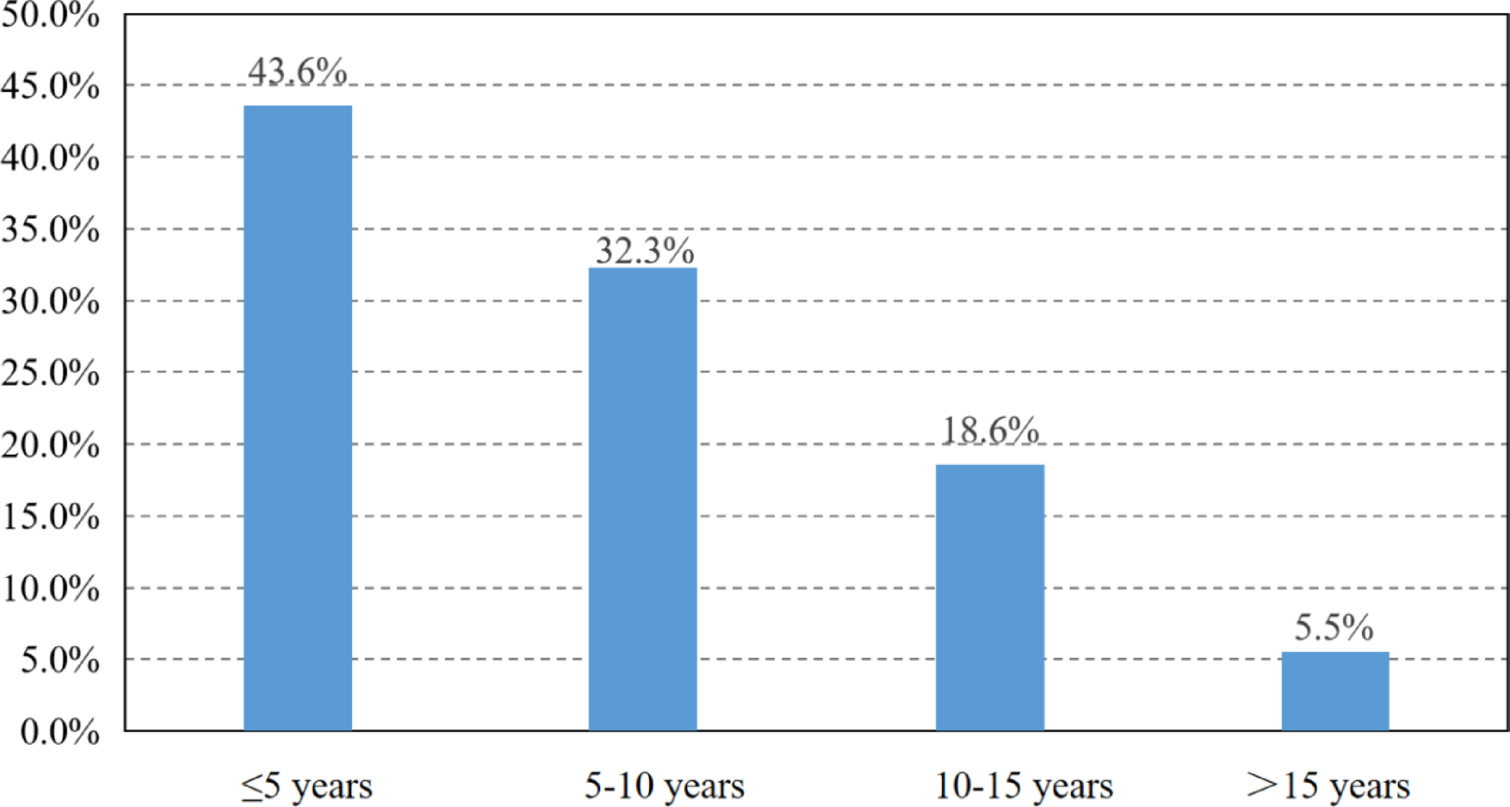
Survival time distribution for breast cancer patients dying of heart disease.

**Fig. 4:**
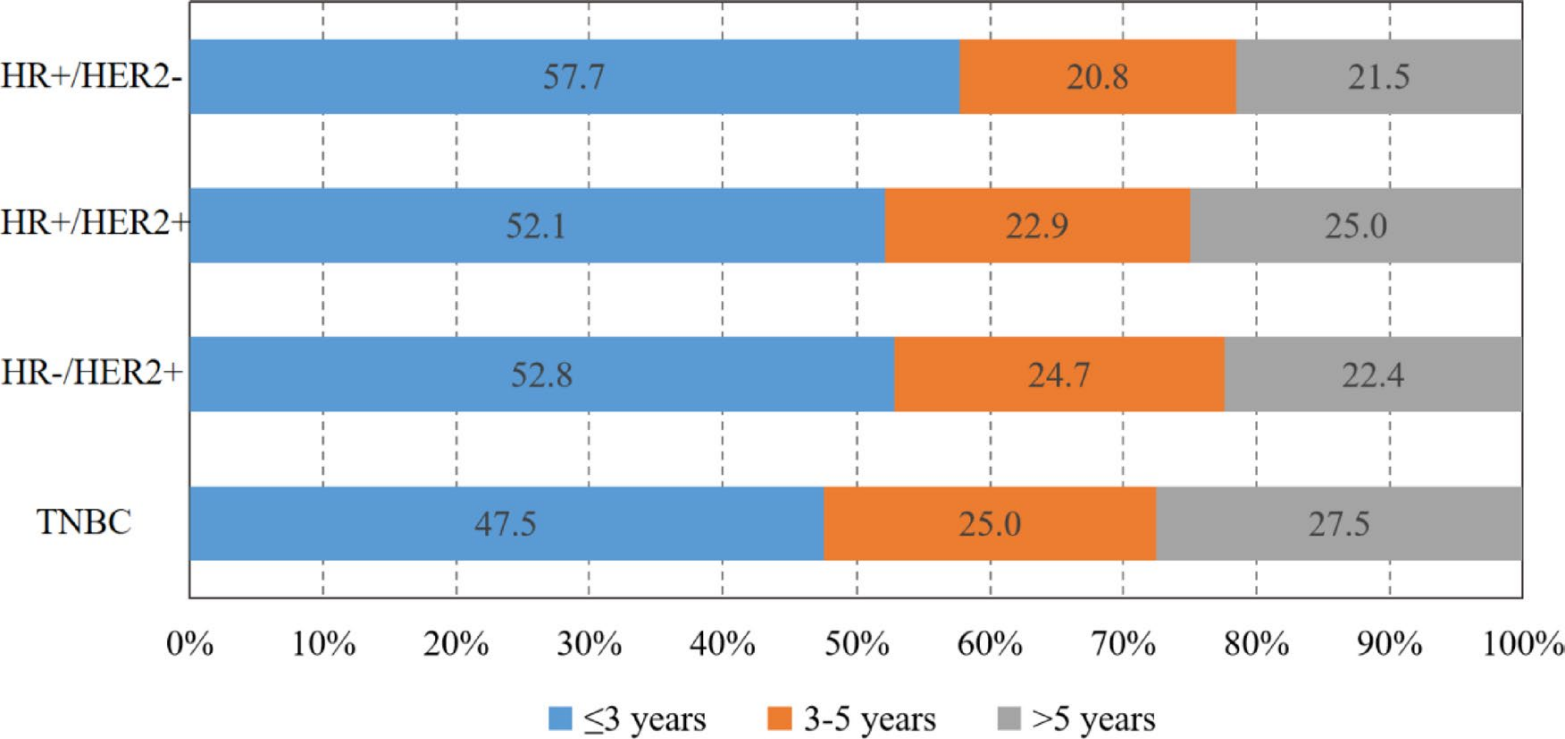
Survival time distribution for breast cancer patients dying of heart disease according to different molecular subtypes. HR, hormone receptor; HER2, human epidermal growth factor receptor 2; TNBC, triple-negative breast cancer.

A total of 655,552 patients with BC met the criteria and were analyzed in our study. After median follow-up of 72 months, 149,708(22.8%)deaths occurred to them. Among the all deaths, 70,512 patients (47.1%) succumbed to breast cancer (BC), with HD being the second cause of mortality, accounting for 22,756 deaths (15.2%). While, with the survival time extending (> 10 years), HD (22.3%) has overtaken BC (20.2%) as the leading cause of death (Fig. 2). Among patients dying of HD, 9922 (43.6%) cases occurred within 5 years after the diagnosis of BC, and 7,350 (32.3%) cases within 5 to 10 years (Fig. 3); higher proportion of patients with TNBC and HER2-positive subtype died within 3 years compared to patients with HR+/HER2- BC (Fig. 4).

### The HSD risk in patients with BC

As shown in Table 1, the overall SMR for HSD was significantly higher in patients with BC compared to the general population (SMR = 8.14, 95% CI: 8.04–8.25). Then, HSD-related SMR were increased in patients diagnosed with BC in 2015–2019 (SMR = 36.52, 95% CI: 34.53–38.59) and patients with younger age (≤ 65 years) (SMR = 67.39, 95% CI: 65.34–69.49). Furthermore, the SMR were also increased in patients with TNBC (SMR = 21.86, 95% CI: 20.05–23.72), HR-/HER2 + subtype (SMR = 29.45, 95% CI: 25.39–33.98) and HR+/HER2 + subtype (SMR = 20.62, 95% CI: 18.81–22.57).

**Table 1:** SMR of heart disease for breast cancer patients.

Variables	All patients (%)	HSD		SMR (95% CI)
		Observed	Expected	
All	655,552	22,718	2790	8.14* (8.04–8.25)
Years of diagnosis				
2000–2004	122,823 (18.74)	1483	399.59	3.71* (3.52–3.91)
2005–2009	152,832 (23.31)	4398	931.38	4.72*(4.58–4.86)
2010–2014	174,482 (26.62)	7074	982.58	7.2* (7.03–7.37)
2015–2019	205,415 (31.33)	9763	475.01	20.55* (20.15–20.96)
Sex				
Female	651,301 (99.4)	22,409	2753.95	8.14* (8.03–8.24)
Male	4251 (0.6)	309	36.05	8.57* (7.64–9.58)
Age (years)				
≤65	428,165 (65.31)	4089	60.68	67.39* (65.34–69.49)
>65	227,387 (34.69)	18,653	2707.55	6.89* (6.79–6.99)
Race				
White	498,875 (76.1)	19,346	2547.55	7.59* (7.49–7.70)
Black	110,788 (16.9)	2323	172.67	13.45* (12.91–14.01)
Other	45,889 (7.0)	1049	69.78	15.03* (14.14–15.97)
Marital status				
Married	384,933 (58.72)	8508	953.97	8.92* (8.73–9.11)
Unmarried	270,619 (41.28)	14,210	1836.03	7.74* (7.61–7.87)
Laterality				
Right	323,260 (49.31)	11,050	1355.53	8.15* (8.00-8.31)
Left	331,770 (50.61)	11,667	1434.47	8.13* (7.99–8.28)
Bilateral	522 (0.08)	1	0.06	16.66* (4.26–127)
Histology				
IDC	492,733 (75.17)	15,787	1864.04	8.47* (8.34–8.60)
ILC	55,352 (8.44)	2158	288.70	7.47* (7.16–7.80)
Others	107,467 (16.39)	4773	637.26	7.49* (7.28–7.71)
ER				
Positive	529,612 (80.79)	19,148	2442.44	7.84* (7.73–7.95)
Negative	125,940 (19.21)	3570	345.56	10.27* (9.94–10.61)
HER2				
Positive	59,346 (9.1)	661	29.32	22.55* (20.86–24.33)
Negative	319,514 (48.7)	4660	284.71	16.37* (15.90-16.84)
NA	276,692 (42.2)	17,397	2475.97	7.03* (6.92–7.13)
Subtype				
HR+/HER2-	277,617 (42.35)	4102	259.15	15.83* (15.35–16.32)
HR+/HER2+	41,949 (6.4)	473	22.94	20.62* (18.81–22.57)
HR-/HER2+	17,397 (2.65)	188	6.38	29.45* (25.39–33.98)
TNBC	41,897 (6.4)	558	25.56	21.83* (20.05–23.72)
NA	276,692 (42.2)	17,397	2475.97	7.03* (6.92–7.13)
Surgery				
BCS	362,959 (55.37)	12,167	1634.39	7.44* (7.31–7.58)
Mastectomy	254,569 (38.83)	9203	1070.94	8.59* (8.42–8.77)
No surgery	38,024 (5.8)	1348	84.68	15.92* (15.08–16.79)
Radiation				
No	300,275 (45.8)	13,902	1667.49	8.34* (8.20–8.48)
Yes	355,277 (54.2)	8816	1122.51	7.85* (7.69–8.02)
Chemotherapy				
No	370,040 (56.45)	18,566	2558.96	7.26* (7.15–7.36)
Yes	285,512 (43.55)	4152	231.04	17.97* (17.43–18.53)

SMR, standard mortality ratios; HSD, heart-specific death; CI, confidence intervals; IDC, invasive ductal carcinoma; ILC, invasive lobular carcinoma; ER, estrogen receptor; HER2, human epidermal growth factor receptor 2; TNBC, triple-negative cancer; NA, not available; HR, hormone receptor.

### Predictors of death from HD in patients with BC

As shown in Table 2, many demographic factors and clinicopathologic characteristics had significant effects on the risk of HSD in patients with BC. Increased risks of HSD were seen in male patients (HR = 2.237, 95%CI: 1.996–2.508) compared with female patients. Then, the older patients (>65 years) had significantly higher risk (HR = 8.784, 95%CI: 8.471–9.110) of HSD compared with younger patients (≤ 65 years). The patients of Black race had slightly increased risk (HR = 1.17, 95%CI: 1.120–1.224) of HSD compared with White race. While, patients of other races had significantly lower risk (HR = 0.657, 95%CI: 0.616–0.699) of HSD than White race. The unmarried patients exhibited significantly higher risk (HR = 1.828, 95%CI: 1.777–1.879) of HSD than married patients. The levels of median household income also had impacts on the risk of HSD for patients with BC, and decreased risks of HSD were seen in patients with median household income of $55,000–75,000 (HR = 0.856, 95%CI: 0.827–0.886) and >$75,000 (HR = 0.785, 95%CI: 0.756–0.815) compared with median household income of less than $55,000. Furthermore, patients with higher grade (II-IV) showed higher risks of HSD compared with patients of grade I. Risks of HSD were seen sightly increased in patients with ER-negative (HR = 1.12, 95%CI: 1.076–1.166) than ER+. Patients with higher tumor stage (III-IV) exhibited significantly higher risks (HR = 1.779, 95%CI: 1.704–1.857) of HSD than patients with lower tumor stage (I-II). Patients with mastectomy showed lower risks of (HR = 1.779, 95%CI: 1.704–1.857) HSD than patients with BCS, but patients without breast surgery showed significantly higher risks of (HR = 1.88, 95%CI: 1.763–2.005) HSD than patients with BCS. The molecular classification of BC also affected the patient’s risk of HSD as shown in Table 3. Compared with HR+/HER2- subtype, higher risks of HSD were seen in patients with TNBC (HR = 1.382, 95%CI: 1.252–1.526) and HR+/HER2- subtype (HR = 1.152, 95%CI: 1.042–1.273).

**Table 2:** Univariate and multivariate analysis of HSD in breast cancer patients.

Variables	Univariate analysis			Multivariate analysis (		
	HR	95% CI	*P*-value	HR	95% CI	*P*-value
Sex						
Female	Ref			Ref		
Male	2.731	2.439–3.057	< 0.001	2.237	1.996–2.508	< 0.001
Age (years)						
≤65	Ref			Ref		
>65	11.819	11.420-12.232	< 0.001	8.784	8.471–9.110	< 0.001
Race						
White	Ref			Ref		
Black	1.132	1.084–1.182	< 0.001	1.17	1.120–1.224	< 0.001
Others	0.489	0.459–0.520	< 0.001	0.657	0.616–0.699	< 0.001
Marital status						
Married	Ref			Ref		
Unmarried	2.817	2.741–2.894	< 0.001	1.828	1.777–1.879	< 0.001
Median household income ($)						
<55,000	Ref			Ref		
55,000–75,000	0.790	0.764–0.817	< 0.001	0.856	0.827–0.886	< 0.001
>75,000	0.648	0.625–0.672	< 0.001	0.785	0.756–0.815	< 0.001
Laterality						
Right	Ref			Ref		
Left	1.026	1.000-1.054	0.053	1.005	0.979–1.032	0.715
Bilateral	1.839	1.044–3.238	0.035	0.528	0.291–0.956	0.035
Histology						
IDC	Ref			Ref		
ILC	1.278	1.220–1.337	< 0.001	1.009	0.962–1.508	0.707
Others	1.228	1.188–1.269	< 0.001	1.053	1.018–1.089	0.003
Grade						
I	Ref			Ref		
II	0.982	0.949–1.016	0.288	1.099	1.061–1.138	< 0.001
III	0.816	0.786–0.847	< 0.001	1.240	1.189–1.293	< 0.001
IV	0.866	0.753–0.995	0.042	1.285	1.117–1.479	< 0.001
unknown	1.221	1.152–1.294	< 0.001	1.119	1.053–1.189	< 0.001
ER						
Positive	Ref			Ref		
Negative	0.810	0.781–0.839	< 0.001	1.12	1.076–1.166	< 0.001
PR						
Positive				Ref		
Negative	0.973	0.945–1.002	0.069	1.078	1.038–1.119	< 0.001
Stage						
I-II	Ref			Ref		
III-IV	1.204	1.159–1.251	< 0.001	1.779	1.704–1.857	< 0.001
Surgery						
BCS	Ref			Ref		
Mastectomy	1.151	1.120–1.183	< 0.001	0.903	0.874–0.934	< 0.001
No surgery	3.314	3.129–3.510	< 0.001	1.88	1.763–2.005	< 0.001

HSD, heart-specific death; CI, confidence intervals; IDC, invasive ductal carcinoma; ILC, invasive lobular carcinoma; ER, estrogen receptor; HER2, human epidermal growth factor receptor 2; NA, not available; HR, hormone receptor; Ref, reference; BCS, breast-conserving surgery.

**Table 3:** Univariate and multivariate analysis of HSD according to molecular subtype.

Variables	Univariate analysis			Multivariate analysis		
	Hazard ratios	95% CI	P-value	Hazard ratios	95% CI	P-value
Subtype						
HR+/HER2-	Ref			Ref		
HR+/HER2+	0.77	0.700-0.847	< 0.001	1.152	1.042–1.273	0.006
HR-/HER2-	0.739	0.637–0.856	< 0.001	1.129	0.966–1.318	0.126
TNBC	0.983	0.900-1.074	0.702	1.382	1.252–1.526	< 0.001

HSD, heart-specific death; CI, confidence intervals; HR, hormone receptor; HER2, human epidermal growth factor receptor 2; TNBC, triple-negative breast cancer; Ref, reference.

## Discussion

CVD and cancer are the leading causes of death in the 21 st century^6,11^, and these two diseases have previously caught much attention from cardiovascular specialists and oncologists, respectively. While, with the improvement of anti-cancer treatment and extending survival for cancer patients, the risk of CVD is becoming more and more serious among cancer patients^10,12^. BC is the most common malignant tumor among women and its long-term survival rate is increasing significantly^1,4,5^, which means more and more BC survivors will be at risk of CVD. Many previous studies have demonstrated that BC patients are more likely to die from CVD than the general population (SMR: 3.78–4.84)^9,10,13^. However, HD is the most serious type of CVD but completely distinct from other CVD. As the results showing in our study, HD (73%) accounts for the majority of BC patients dying from CVD. Furthermore, the overall SMR (8.14) for HSD was significantly higher in patients with BC compared to the general population. These indicated that patients with BC should pay more attention to HD than other CVD. In our study, the majority of deaths from HD occurred within 10 years after BC diagnosis, which was more pronounced in patients with TNBC and HER2 + BC (within 3 years). This suggests that pathological factors and anti-cancer treatments may influence the risk of death from HD for patients with BC and attention should be paid to preventing HD during the treatment of breast cancer a few years after the diagnosis of BC.

Previous studies have shown that many demographic characteristics will influence the incidence and mortality of HD in the general population, which was also seen in patients with BC. Men have a higher incidence of HD and are more likely to die from HD than women in the general population^14,15^. In our study, male presented more than twice the risk of dying from HD compared to female in patients with BC. Although BC is a rare disease for men, more attention should be paid to the prevention and treatment of HD when BC happening to men. Old age is widely recognized as a risk factor for HD and older patients are also more likely to die from HD in the general population^16^. Also, older patients significantly presented higher risk of HSD compared to younger patients in our study, which suggests that the prevention and treatment of HD should be focused on the older patients with BC. Then, the Black race exhibited higher risk of mortality from HD in our study, which was the same as the general population reported by previous studies^17,18^. Some studies have attributed this difference to socioeconomic factors, including income, access to health care, education level and living environment^19,20^. Individuals from other racial groups, including American Indian/Alaska Native and Asian/Pacific Islander, exhibited higher SMRs compared to the White population. However, after adjusting for confounders through multivariate analysis, a lower risk of HSD was observed among these groups compared to White individuals. This inconsistency underscores the elevated SMR observed in other races, which is likely driven by systemic disparities in healthcare access and delays in diagnosis at later stages. Furthermore, marital status also had a significant impact on the risk of death from HD in BC patients, the unmarried patients showed higher risk of death from HD than the married patients in our study. Previous studies have shown that married people have better heart health^21^. However, the unmarried people are more likely to be socially isolated, which significantly increases the risk of death from HD^22^. Meanwhile, developing BC is a devastating strike to the psychological health of many patients^23^, especially for patients undergoing mastectomy^24^. Therefore, unmarried status may aggravate the adverse psychological problems of breast cancer patients, which further contributes to the death from HD in patients with BC. Previous researches demonstrated that household income also had a significant impact on the risk of HD, and the population with higher household income exhibited a lower risk of genetic susceptibility to myocardial infarction and hypertension^25^. In our study, the patients with higher median household income showed decreasing risk of death from HD compared with the lower median household income (<$55000). Higher household income families have better access to quality medical services, including regular health checkups and disease prevention measures, which can help in the early detection and management of heart disease risk factors. Also, higher-income groups tend to have better conditions and resources to maintain a healthy lifestyle, such as a healthier diet, more physical activity, and avoiding undesirable addictions (such as smoking and excessive alcohol consumption)^26^. These lifestyle factors significantly reduced the incidence of heart disease. Thus, low-income BC patients should pay more attention to the prevention of HD after the diagnosis of BC.

In addition to the above demographic characteristics, the clinicopathological features of BC can also influence the risk of death from HD. Increased risk of death from HD were seen in patients with higher grade, negative ER/PR status, HER2 overexpression, TNBC subtype and later tumor stage in our study. These pathological features often indicate a higher degree of malignancy. Actually, the occurrence of tumor can cause a series of immune responses in the body, and induce the production of pro-inflammatory cytokines, such as tumor necrosis factor-α (TNF-α), interleukin-1β (IL-1β), interleukin-6 (IL-6)^27^. The levels of those pro-inflammatory cytokines are positively correlated with the malignancy and stage of the BC^28,29^. Furthermore, those pro-inflammatory cytokines may damage vascular endothelial cells and worse the heart disease by accelerating the progression of coronary atherosclerosis^30–32^. Thus, patients with higher degree of malignancy and later tumor stage may have more pro-inflammatory cytokines in their circulatory system, which further increases the risk of death from HD. Nevertheless, more rigorous and in-depth researches are needed to confirm our hypothesis. In addition to the pro-inflammatory factors producing from tumors, aggressive anti-cancer treatments may also accelerate the death of HD in patients with larger tumor burden. Stronger chemotherapy drugs are often administrated to those patients, and increasing trends in HD mortality were observed among regional-stage patients who received chemotherapy^33^. Actually, many chemotherapy drugs are toxic to the heart and can cause severe heart damages. Especially doxorubicin, commonly used for breast cancer, can cause severe irreversible heart damage, which is positively correlated with cumulative dose^34,35^. Then, previous studies have shown that trastuzumab can lead to decreased heart function and heart failure in patients with HER2-positive BC^36,37^, especially when used in combination with doxorubicin^38^, which may be the reason for the higher risk of death from HD in HER2-positive breast cancer in our study. Furthermore, radiotherapy is usually an indispensable treatment for patients with large tumors or lymph node metastases, which can also cause serious heart damages, such as coronary artery disease or cardiomyopathy^39^, resulting in increased rates of percutaneous coronary intervention (PCI) and reduced survival outcomes^40^. Surprising, patients who did not undergo surgery had a higher risk of death from HD compared with those who received breast-conserving surgery, which can be attributed to several factors. Firstly, patients who did not receive surgery often had poorer baseline health, including advanced age and multiple comorbidities, which increased their risk of HD. Secondly, pre-existing HD may have been a contraindication for surgery, leading to non-surgical treatment options. Additionally, the bi-directional relationship between HD and BC treatment, such as chemotherapy and radiation, can exacerbate cardiac conditions. This highlights the need for a multidisciplinary approach in managing these patients, emphasizing comprehensive risk assessment and personalized treatment strategies.

Based on our above research findings, breast cancer patients may consider the following measures to reduce the risk of death from HD. First, lifestyle modifications, such as adopting a heart-healthy diet, engaging in regular physical activity, and quitting smoking, are crucial for reducing cardiovascular risk. Additionally, optimizing the management of comorbid conditions like hypertension and diabetes is essential. Moreover, a multidisciplinary care model involving oncologists, cardiologists, and primary care providers can ensure comprehensive management of both cancer and cardiovascular health.

Several limitations of this study must be clarified. Firstly, some bias is inevitable due to the retrospective nature of our study. Secondly, the risk factors for HD can not be acquired in the SEER database, such as hypertension, serum cholesterol level, smoking, diabetes, obesity, amount of exercise and unhealthy diet. Third, the history of heart disease and specific cause of heart disease are also not available in the SEER database. Furthermore, the specific treatment information is also not available from the database, such as endocrine therapy, anti-HER2 targeted therapy and detailed chemotherapy regimens, which may also contribute to the HD. Nevertheless, our study confirmed that BC patients have a significantly increased risk of dying from HD compared to the general population with a larger sample size. Also, we estimated the risk factors for death of HD in patients with BC, which may help clinicians reduce HD in BC patients.

## Conclusion

The risk of HSD was significantly higher in BC patients than the general population, and occurred primarily within 10 years after BC diagnosis. Demographic characteristics and tumor clinicopathological factors have impacts on the risk of HSD in BC patients. Medical strategies are needed to reduce the risk of HD among patients with BC.

## Data Availability

The datasets presented in this study can be found in online repository: the Surveillance, Epidemiology, and End Results (SEER) database (https://seer.cancer.gov).

## References

[1] Sung, H. et al. Global Cancer statistics 2020: GLOBOCAN estimates of incidence and mortality worldwide for 36 cancers in 185 countries. *CA Cancer J. Clin.* **71** (3), 209–249 (2021). 33538338 10.3322/caac.21660

[2] Wockel, A. et al. The screening, diagnosis, treatment, and Follow-Up of breast Cancer. *Dtsch. Arztebl Int.* **115** (18), 316–323 (2018). 29807560 10.3238/arztebl.2018.0316 PMC5987060

[3] Caswell-Jin, J. L. et al. Analysis of breast Cancer mortality in the US-1975 to 2019. *JAMA* **331** (3), 233–241 (2024). 38227031 10.1001/jama.2023.25881 PMC10792466

[4] Zitricky, F. et al. Conditional survival in breast cancer up to 10 years in the nordic countries. *Cancer Med.* **12** (17), 17945–17951 (2023). 37578395 10.1002/cam4.6436 PMC10524006

[5] Liu, G. et al. Clinical features and prognoses of patients with breast Cancer who underwent surgery. *JAMA Netw. Open.* **6** (8), e2331078 (2023). 37624596 10.1001/jamanetworkopen.2023.31078 PMC10457722

[6] Roth, G. A. et al. Global burden of cardiovascular diseases and risk factors, 1990–2019: update from the GBD 2019 study. *J. Am. Coll. Cardiol.* **76** (25), 2982–3021 (2020). 33309175 10.1016/j.jacc.2020.11.010 PMC7755038

[7] Abdel-Qadir, H. et al. A Population-Based study of cardiovascular mortality following Early-Stage breast Cancer. *JAMA Cardiol.* **2** (1), 88–93 (2017). 27732702 10.1001/jamacardio.2016.3841

[8] Patnaik, J. L. et al. Cardiovascular disease competes with breast cancer as the leading cause of death for older females diagnosed with breast cancer: a retrospective cohort study. *Breast Cancer Res.* **13** (3), R64 (2011). 21689398 10.1186/bcr2901 PMC3218953

[9] He, J. et al. Competing risk analysis of cardiovascular death in breast cancer: evidence from the SEER database. *Transl Cancer Res.* **12** (12), 3591–3603 (2023). 38192997 10.21037/tcr-23-1163 PMC10774043

[10] Sturgeon, K. M. et al. A population-based study of cardiovascular disease mortality risk in US cancer patients. *Eur. Heart J.* **40** (48), 3889–3897 (2019). 31761945 10.1093/eurheartj/ehz766 PMC6925383

[11] Bray, F. et al. The ever-increasing importance of cancer as a leading cause of premature death worldwide. *Cancer* **127** (16), 3029–3030 (2021). 34086348 10.1002/cncr.33587

[12] Armenian, S. H. et al. Cardiovascular disease among survivors of Adult-Onset cancer: A Community-Based retrospective cohort study. *J. Clin. Oncol.* **34** (10), 1122–1130 (2016). 26834065 10.1200/JCO.2015.64.0409 PMC7357493

[13] Wang, D. et al. Cause-specific mortality among patients with different molecular subtypes of T1-2N0M0 breast cancer: A population-based study. *Med. (Baltim).* **100** (43), e27605 (2021). 10.1097/MD.0000000000027605 PMC8556021 34713838

[14] Menotti, A. & Puddu, P. E. Epidemiology of heart disease of uncertain etiology: A population study and review of the problem. *Medicina (Kaunas)* **55**(10), 687 (2019). 31615121 10.3390/medicina55100687 PMC6843161

[15] Qiu, W. et al. Sex difference in incidence and risk factors of hospitalization for heart failure, and subsequent mortality: findings from the China PEACE million persons project. *BMC Public. Health*. **23** (1), 2356 (2023). 38017495 10.1186/s12889-023-17286-z PMC10685651

[16] Wei, D. et al. Age-Period-Cohort analysis of ischemic heart disease morbidity and mortality in china, 1990–2019. *Circ. J.* **86** (9), 1437–1443 (2022). 35569970 10.1253/circj.CJ-21-0749

[17] Heron, M. Deaths: leading causes for 2019. *Natl. Vital Stat. Rep.* **70** (9), 1–114 (2021). 34520342

[18] Deo, R. et al. Differences in risk of sudden cardiac death between Blacks and Whites. *J. Am. Coll. Cardiol.* **72** (20), 2431–2439 (2018). 30442286 10.1016/j.jacc.2018.08.2173 PMC9704756

[19] Levine, R. S. et al. Black-white inequalities in mortality and life expectancy, 1933–1999: implications for healthy people 2010. *Public. Health Rep.* **116** (5), 474–483 (2001). 12042611 10.1093/phr/116.5.474 PMC1497364

[20] Graham, G. Disparities in cardiovascular disease risk in the united States. *Curr. Cardiol. Rev.* **11** (3), 238–245 (2015). 25418513 10.2174/1573403X11666141122220003 PMC4558355

[21] Manfredini, R. et al. Marital status, cardiovascular diseases, and cardiovascular risk factors: A review of the evidence. *J. Women’s Health*. **26** (6), 624–632 (2017). 10.1089/jwh.2016.6103 28128671

[22] Holt-Lunstad, J., Smith, T. B. & Layton, J. B. Social relationships and mortality risk: a meta-analytic review. *PLoS Med.* **7** (7), e1000316 (2010). 20668659 10.1371/journal.pmed.1000316 PMC2910600

[23] Parikh, D. et al. Post-traumatic stress disorder and Post-traumatic growth in breast Cancer Patients - a systematic review. *Asian Pac. J. Cancer Prev.* **16** (2), 641–646 (2015). 25684500 10.7314/apjcp.2015.16.2.641

[24] Hanson, S. E. et al. Long-term quality of life in patients with breast Cancer after breast conservation vs mastectomy and reconstruction. *JAMA Surg.* **157**(6), e220631 (2022). 35416926 10.1001/jamasurg.2022.0631 PMC9008558

[25] Zheng, X. et al. Dissecting the causal relationship between household income status and genetic susceptibility to cardiovascular-related diseases: insights from bidirectional Mendelian randomization study. *BMC Public. Health* **23**(1), 747 (2023). 37095467 10.1186/s12889-023-15561-7 PMC10124030

[26] Zhang, X. et al. Healthy lifestyle behaviours and all-cause and cardiovascular mortality among 0.9 million Chinese adults. *Int. J. Behav. Nutr. Phys. Act.* **18**(1), 162 (2021). 34922591 10.1186/s12966-021-01234-4 PMC8684211

[27] Habanjar, O. et al. Crosstalk of inflammatory cytokines within the breast tumor microenvironment. *Int. J. Mol. Sci.* **24**(4), 4002 (2023). 36835413 10.3390/ijms24044002 PMC9964711

[28] Sheen-Chen, S. M. et al. Serum concentration of tumor necrosis factor in patients with breast cancer. *Breast Cancer Res. Treat.* **43** (3), 211–215 (1997). 9150900 10.1023/a:1005736712307

[29] Ma, Y. et al. IL-6, IL-8 and TNF-alpha levels correlate with disease stage in breast cancer patients. *Adv. Clin. Exp. Med.* **26** (3), 421–426 (2017). 28791816 10.17219/acem/62120

[30] Wu, Y. et al. The expression of SAH, IL-1β, hcy, TNF-α and BDNF in coronary heart disease and its relationship with the severity of coronary stenosis. *BMC Cardiovasc. Disord.* **22**(1), 101 (2022). 35282820 10.1186/s12872-021-02388-6 PMC8919521

[31] Bevilacqua, M. P. et al. Interleukin-1 activation of vascular endothelium. Effects on procoagulant activity and leukocyte adhesion. *Am. J. Pathol.* **121** (3), 394–403 (1985). 3878084 PMC1887931

[32] Cai, T. et al. Association of Interleukin 6 receptor variant with cardiovascular disease effects of Interleukin 6 receptor blocking therapy. *JAMA Cardiol.* **3**(9), 849–857 (2018). 30090940 10.1001/jamacardio.2018.2287 PMC6233652

[33] Vo, J. B. et al. Trends in heart disease mortality among breast cancer survivors in the US, 1975–2017. *Breast Cancer Res. Treat.* **192** (3), 611–622 (2022). 35107712 10.1007/s10549-022-06515-5 PMC8960573

[34] Swain, S. M., Whaley, F. S. & Ewer, M. S. Congestive heart failure in patients treated with doxorubicin: a retrospective analysis of three trials. *Cancer* **97** (11), 2869–2879 (2003). 12767102 10.1002/cncr.11407

[35] Qiu, Y., Jiang, P. & Huang, Y. Anthracycline-induced cardiotoxicity: mechanisms, monitoring, and prevention. *Front. Cardiovasc. Med.* **10**, 1242596 (2023). 38173817 10.3389/fcvm.2023.1242596 PMC10762801

[36] Ewer, S. M. & Ewer, M. S. Cardiotoxicity profile of trastuzumab. *Drug Saf.* **31** (6), 459–467 (2008). 18484781 10.2165/00002018-200831060-00002

[37] Ewer, M. S. et al. Reversibility of trastuzumab-related cardiotoxicity: new insights based on clinical course and response to medical treatment. *J. Clin. Oncol.* **23** (31), 7820–7826 (2005). 16258084 10.1200/JCO.2005.13.300

[38] Perez, E. A. et al. Cardiac safety analysis of doxorubicin and cyclophosphamide followed by Paclitaxel with or without trastuzumab in the North central Cancer treatment group N9831 adjuvant breast cancer trial. *J. Clin. Oncol.* **26** (8), 1231–1238 (2008). 18250349 10.1200/JCO.2007.13.5467 PMC4048960

[39] Taunk, N. K. et al. Radiation-induced heart disease: pathologic abnormalities and putative mechanisms. *Front. Oncol.* **5**, 39 (2015). 25741474 10.3389/fonc.2015.00039 PMC4332338

[40] Boero, I. J. et al. Modern radiation therapy and cardiac outcomes in breast Cancer. *Int. J. Radiat. Oncol. Biol. Phys.* **94** (4), 700–708 (2016). 26972642 10.1016/j.ijrobp.2015.12.018

